# Glucocorticoids: an enemy or a friend for the dermatologist? recommendations for proper use and safe discontinuation

**DOI:** 10.1007/s12020-026-04778-2

**Published:** 2026-09-16

**Authors:** Evangelia Sali, Ioanna A. Paschou, Sofia M. Dimopoulou, Anna Tagka, Vasiliki Chasapi, Electra Nicolaidou, Alexander J. Stratigos, Melpomeni Peppa, Stavroula A. Paschou

**Affiliations:** 1 Endocrine Unit and Diabetes Centre, Department of Clinical Therapeutics, Alexandra Hospital, School of Medicine, National and Kapodistrian University of Athens, Athens, Greece; 2 Department of Dermatology and Venereology, Andreas Sygros Hospital, School of Medicine, National and Kapodistrian University of Athens, Athens, Greece; 3 Endocrine Unit, Second Department of Internal Medicine, Attikon University Hospital, School of Medicine, National and Kapodistrian University of Athens, Athens, Greece; 4 Endocrine Unit, Third Department of Internal Medicine, Sotiria General Hospital, School of Medicine, National and Kapodistrian University of Athens, Athens, Greece; 5 Imperial Centre for Endocrinology, St Mary’s Hospital, Imperial College London, London, UK

**Keywords:** Glucocorticoid, Skin Diseases, Dermatology, Adrenal Insufficiency, HPA axis, Hydrocortisone

## Abstract

**Purpose:**

Glucocorticoids (GCs) are steroid hormones produced by the adrenal cortex with anti-inflammatory and immunoregulating effects, thus essential for the management of numerous inflammatory and autoimmune disorders, including a wide range of dermatological conditions. The aim of our review is to summarize current data about the indications for GCs in dermatology, present the dermatological and endocrinological adverse effects and summarize current recommendations about the proper use and safe discontinuation of GCs, creating a practical guide for dermatologists.

**Methods:**

A narrative review of the literature on PubMed/MEDLINE was conducted and included articles in English, published until July, 2026. A combination of the keywords “glucocorticoids”, “dermatology”, “adrenal insufficiency”, “HPA axis”, “adverse effects” and “monitoring” was used to identify relevant literature for this review, with emphasis on recent recommendations and guidelines. The reference lists of the selected publications were also reviewed.

**Results:**

Although GCs are commonly prescribed medications in dermatology, their use is associated with numerous adverse effects, extending beyond the skin to multiple organ systems. However, some side effects, such as adrenal insufficiency and withdrawal syndrome, may even be life-threatening.

**Conclusion:**

Due to the expanded use of GCs, careful prescription and safe discontinuation is required. For this reason, we have created a practical clinical guide, summarizing the relevant recommendations to ensure proper use of GCs as well as safe discontinuation of the treatment. Optimal GC use requires individualized and careful selection of potency, duration, and route of administration to prevent any side effects and ensure the best outcome for the patient.

## Introduction

Glucocorticoids (GCs) are synthetic analogues of the endogenous hormones produced by the adrenal cortex under the hypothalamic–pituitary–adrenal (HPA) axis control [1]. GCs act on multiple tissues by binding to intracellular glucocorticoid receptors (GR) and regulating the expression of various genes involved in the immune response [1–3]. GCs have significant effects on the HPA axis through negative feedback, as GCs can bind to GR and inhibit the expression of the corticotropin-releasing hormone (CRH) and the adrenocorticotropic hormone (ACTH) [3, 4].

There are many synthetic GCs with different potencies, half-life times, and routes of administration [1, 3, 5]. Topical GCs are categorized by the European GCs classification system into class I-IV according to their potency, with class I representing low potency GCs and class IV very high potency GCs (Table 1**)**. A seven-class system of GCs potency is also widely used [6, 7]. The equivalent doses, duration of action and potency of systemic GCs are presented in Table 2. Hydrocortisone is mainly used as a replacement therapy, while the other agents are primarily used for their anti-inflammatory effects and can be used in many medical specialties [8].

**Table 1:** Potency classes of topical GCs

GCs potency classes	Representative agents	Suggested anatomical sites and types of skin lesions
**Class I – Low**	Hydrocortisone acetate 1% Methylprednisolone acetate 0.25%	Areas with thin epidermis, such as face, eyelids, neck and genital area Large surface area of the body in need of treatment
**Class II – Medium**	Clobetasone butyrate 0.05% Fluocortolone pivalate 0.5%	Acute inflammatory lesions in the trunk and extremities
**Class III – High**	Betamethasone valerate 0.1% Fluocinolone acetonide 0.025% Fluticasone propionate 0.05% Mometasone furoate 0.1%	Chronic inflammatory lesions in the trunk and extremities
**Class IV – Very High**	Clobetasol propionate 0.05% Halcinonide 0.01%	Areas with thick skin, such as the palms and soles Hyperkeratotic lesions

The table presents the European classification of topical GCs’ potency, according to which GCs are divided into four classes, from least to most potent. For each class, there are some representative agents presented, as well as the suggested anatomical site and types of lesions suitable [6, 7]

**Table 2:** GCs equivalent doses for systemic administration

Glucocorticoids	Equivalent dose	Route of administration	Potency	Duration of action
Hydrocortisone	20 mg	PO / IV	Low	Short acting (8–12 h)
Cortisone	25 mg	PO	Low	Short acting (8–12 h)
Prednisone	5 mg	PO	Moderate	Intermediate acting (12–36 h)
Prednisolone	5 mg	PO	Moderate	Intermediate acting (12–36 h)
Methylprednisolone	4 mg	PO / IV	Moderate	Intermediate acting (12–36 h)
Dexamethasone	0.5 mg	PO / IV	High	Long acting (36–72 h)
Betamethasone	0.5–0.75 mg	PO / IM / IV	High	Long acting (36–72 h)

The table presents commonly prescribed GCs, the equivalent doses, the route of administration for each one, and categorizes the GCs based on the potency and duration of action [1, 8]

The extended use of GCs highlights the importance of their correct use, the appropriate prevention measures, as well as the management of the possible side effects. Although there are multiple published recommendations and guidelines about the use of GCs, there is a lack of dermatology–centered practical points. In our review, not only do we summarize the current recommendations, but we also focus on the practical and clinically important aspects for dermatologists regarding GCs as a therapeutic approach.

### Dermatological indications for GCs administration

GCs are commonly prescribed medications for many dermatological conditions. Topical treatment can be used in different formulations, such as creams, lotions, ointments, gels and sprays [6, 9]. Topical GCs constitute a fundamental therapeutic approach in dermatitis, papulosquamous dermatoses, bullous skin diseases and the cutaneous manifestations of connective tissue disorders and vasculitis [10–28]. Systemic administration of GCs, oral or intravenous, is suggested for conditions requiring rapid and substantial suppression of immune activity, such as autoimmune, bullous and systemic diseases with cutaneous involvement [18, 19, 21, 29, 30]. The dermatological indications for the use of GCs in dermatology are presented in Table 3.

**Table 3:** Dermatological indications for GC treatment

Dermatitis	Neutrophilic dermatoses
• Atopic dermatitis	• Sweet syndrome
• Seborrheic dermatitis	• Behçet disease
• Contact dermatitis	• Pyoderma gangrenosum
**Papulosquamous dermatoses**	• Neutrophilic eccrine hidradenitis
• Psoriasis	**Connective tissue disorders**
• Lichen planus	• Systemic lupus erythematosus
• Pityriasis	• Dermatomyositis
**Bullous skin diseases**	• Sjögren’s syndrome
• Pemphigus	**Vasculitis**
• Bullous pemphigoid	• Cutaneous IgM/IgG immune complex vasculitis
**Granulomatous disorders**	• Skin-limited IgA vasculitis
• Granuloma annulare	• Cutaneous polyarteritis nodosa
• Sarcoidosis	• Urticarial vasculitis
**Alopecia areata**	**Severe hypersensitivity reactions**
**Lichen sclerosus**	• Acute generalised exanthematous pustulosis (AGEP)
**Vitiligo**	• Drug reaction with eosinophilia and systemic symptoms (DRESS)

The table presents a list of common dermatological conditions where GCs are indicated as a treatment option [10–30]. The choice between topical or systemic treatment depends on the specific disease, as well as the severity or requirement for rapid immunosuppression

However, according to current therapeutic approaches, GCs are rarely used as monotherapy, due to the expanding use of biologics and targeted immunoregulatory agents. This approach significantly reduces the need for prolonged systemic GCs therapy and the risk of serious corticosteroid-related adverse effects.

### Adverse effects

GCs treatment may have severe side effects, some of them even life-threatening. Although side effects are more common in patients receiving high doses of GCs for prolonged periods of time, they can occur in any patient. The most prevalent adverse effects are fractures and osteoporosis, cardiovascular disease, while less studied outcomes include diabetes mellitus (DM), gastrointestinal events, and ocular disorders, such as glaucoma [31–33]. Chronic administration of GCs can also lead to immunosuppression and a higher risk of opportunistic infections, due to the reduced migration and increased apoptosis of immune cells [34]. The most common side effects are presented in Table 4.

**Table 4:** Adverse effects of GCs

Dermatological adverse effects	Skin atrophy • Telangiectasia • Striae distensae • Purpura • Stellate pseudoscars • Ulceration Cutaneous infections • Tinea incognito • Secondary bacterial infections Acne Rosacea Perioral dermatitis Hypopigmentation /Hyperpigmentation Folliculitis Skin barrier dysfunction
**Endocrinological adverse effects**	Cushing syndrome • Facial plethora (moon face) • Dorsocervical fat pad hypertrophy (buffalo hump) • Visceral obesity • Striae rubrae Metabolic syndrome • Insulin resistance • Dyslipidemia Adrenal insufficiency • Hypotension • Fatigue • Weight loss • Syncope • Gastrointestinal symptoms • Hyponatremia and hypoglycemia Withdrawal syndrome • Weakness and fatigue • Nausea • Myalgias and arthralgias • Depression or irritability • Cushingoid features Adrenal crisis • Hypotension or shock • Altered mental state • Abdominal pain • Nausea and vomiting • Electrolyte disturbances
**Cardiovascular adverse effects**	Hypertension Fluid retention and edema Venous thromboembolism
**Musculoskeletal adverse effects**	Osteoporosis Myopathy Avascular necrosis
**Neuropsychiatric adverse effects**	Mania and hypomania Psychosis, confusion and delirium Insomnia Behavioral changes and mood disorders
**Ocular adverse effects**	Ocular hypertension Glaucoma Cataract
**Immunosuppression**	Pneumocystis jirovecii pneumonia Herpes zoster Hepatitis B Tuberculosis

The table presents some of the side effects related to topical or systemic administration of GCs and the clinical presentation of those conditions [5, 8], 31– [43]

### Dermatological adverse effects

Topical GCs have been documented to cause side effects in the area in which they are applied. The most common adverse reaction to topical GCs is skin atrophy. This is the result of reduced collagen and mucopolysaccharide production from dermal fibroblasts, impaired keratinocyte and fibroblast proliferation and is presented with telangiectasias, striae distensae, purpura, stellate pseudoscars and ulcerations [33, 35]. Acne exacerbation is another side effect of topical GCs. Although GCs are anti-inflammatory medications, they have been shown to increase TLR2 expression, which gets stimulated by C.Acnes. The same mechanism may be responsible for rosacea and perioral dermatitis due to GCs [36]. Other side effects include hypopigmentation, folliculitis, and skin barrier dysfunction, while prolonged application to the periorbital area may increase the risk of glaucoma and cataract development [33, 35, 37]. Furthermore, GCs can alter the clinical manifestation of infections, resulting in tinea incognito, and lead to secondary bacterial infections or exacerbation of cutaneous infections [35, 38]. Skin-related adverse effects can also occur with systemic GCs administration and are associated with exogenous GCs excess (iatrogenic Cushing’s syndrome) [39, 40].

### Endocrinological adverse effects

Exogenous administration of GCs can lead to iatrogenic Cushing’s syndrome, the most common form of the syndrome [39]. This is possible with systemic, but also with topical treatment, especially in children due to their higher total body surface area to weight, or when the treatment is applied in areas of the body with thin skin [35]. Cushing’s syndrome presents with facial plethora, dorsocervical fat pad hypertrophy (buffalo hump), visceral obesity and purple striae [40]. Other complications include hyperglycemia, insulin resistance, cardiovascular disease, osteoporosis, myopathy, menstrual irregularities, sleep disturbances, mood changes, and psychiatric symptoms [5, 39, 41].

Lastly, prolonged GCs therapy can suppress the HPA axis, leading to GC-induced adrenal insufficiency (AI). The exogenous administration of GCs can inhibit CRH and ACTH production through negative feedback, and thus, reduce the endogenous production of cortisol [4, 8]. HPA axis suppression can be a side effect of GCs treatment by any route of administration and can develop either after the discontinuation of treatment with supraphysiologic doses of GCs, or because of increased GC requirements, both leading to the development of AI [4, 8, 42]. The clinical presentation includes hypotension, fatigue, weight loss, syncope and gastrointestinal symptoms, while biochemical evaluation may reveal hyponatremia and hypoglycemia. Patients with AI may also be asymptomatic or develop life-threatening adrenal crisis [8, 43]. Withdrawal syndrome due to GCs can also be present in patients when supraphysiologic doses are tapered. Symptoms of withdrawal include weakness, fatigue, nausea, myalgias, arthralgias, depression, irritability, or Cushingoid features [8].

### Recommendations for the proper use of GCs, discontinuation of the therapy, and endocrinological evaluation

The administration of GCs, whether as topical or systemic treatment, requires careful assessment of any potential contraindications, as their inappropriate use may exacerbate pre-existing conditions or lead to serious complications. Absolute contraindications to systemic GCs administration include active or uncontrolled infections, particularly systemic fungal infections, severe bacteremia or sepsis, and severe immunosuppression [44–46]. Some relative contraindications are diabetes mellitus, hypertension, heart failure, osteoporosis, peptic ulcer, glaucoma, and psychiatric disorders [44, 45]. For those patients, GCs should be administered in adjusted doses, while close clinical monitoring is essential. Topical GCs treatment is contraindicated in case of active bacterial, viral, or fungal infection, as corticosteroids may exacerbate or mask the clinical manifestation of the infection [6].

Topical GCs are typically applied once or twice daily. Low-potency agents are preferred for areas with thin skin, such as the face, skin folds, and the genital area, whereas higher-potency GCs are preferred in areas with thicker skin, such as the palms and soles [6, 33, 35, 37]. The selection of GCs’ potency is also based on the targeted dermatological condition. Acute inflammatory skin diseases are most often treated with low or moderate potency GCs, while high potency GCs are more effective in chronic inflammations and hyperkeratosis [35]. In long-term treatments with GCs, lower potency agents, intermittent therapy, or a combination therapy with emollients and non-steroidal immunomodulatory agents is preferred [6].

The recommendations regarding systemic GCs are to administer the lowest effective dose for the shortest possible duration. Also, long-acting GCs should be used only when it is required and should be switched to short-acting GCs [8]. GCs should also be administered in morning doses, according to the cortisol circadian rhythm [5].

Patients treated with GCs should be monitored for the course of the treatment and should be educated on the early signs of the possible side effects. With the initiation of the treatment, patients’ blood pressure, lipid profile and cardiovascular risk should be evaluated [29, 44]. The lipid profile should be reassessed after a month of treatment initiation, and then annually [44]. Patients should also be monitored for GC-induced hyperglycemia, especially high-risk patients or those with pre-existing hyperglycemia or diabetes. After the initiation of the treatment, they should be advised to test their blood glucose levels daily or four times a day if they suffer from diabetes [29, 44, 47, 48]. Elevated postprandial blood glucose levels or a pathological oral glucose tolerance test sets the diagnosis of GC-induced hyperglycemia [47, 48]. Every patient treated with GCs for more than three months should also be monitored for GC-induced osteoporosis and be prescribed vitamin D and calcium [49–51]. The osteoporosis assessment includes monitoring of height and weight, a complete history of fractures, the use of the FRAX tool, measurement of bone density and biochemical assessment [50, 51]. Finally, screening for latent tuberculosis should be performed using a chest X-ray and either an interferon-gamma release assay or a tuberculin skin test. Screening for infectious and chronic diseases such as hepatitis B and C, HIV, and varicella zoster virus is also recommended. For patients receiving high-dose GCs for more than 2–4 weeks, prophylaxis against Pneumocystis jirovecii pneumonia is advised [5, 52].

The chronic exogenous administration of corticosteroids at supraphysiological levels can suppress the physiological secretion of ACTH, leading to atrophy of the adrenal cortex [8, 43]. As a result, there is a risk of developing adrenal crisis after the reduction of the dose or the discontinuation of the treatment. At the same time, patients with suspected or established AI being treated with physiological doses of GCs should be educated on “sick-day rules” in instances of increased stress, to prevent a life-threatening adrenal crisis. Those recommendations, “sick-day rules”, suggest that in case of illness, patients should double the dose of GCs. Serious illnesses, intense diarrhea or vomiting dictate that patients be referred to the nearest hospital to receive 100 mg of hydrocortisone IV and fluids quickly, followed by 200 mg of continuous hydrocortisone infusion [8, 44, 53]. Furthermore, patients with established AI should also carry with them an emergency card indicating the management of adrenal crisis, and a hydrocortisone emergency injection kit [8, 53]. Surgical stress also requires increased doses of GCs. In minor surgical procedures, 25–75 mg of IV hydrocortisone can be administered, while in major procedures, 100 mg of hydrocortisone IV, followed by 200 mg of continuous infusion over 24 h, should be given [8, 44, 49, 54, 55]. Patients on supraphysiologic doses of GCs do not require stress dose coverage; however, they can develop adrenal crisis in case of decreased GCs availability, more commonly due to extensive diarrhea, vomiting or a missed dose [8].

**Fig. 1:**
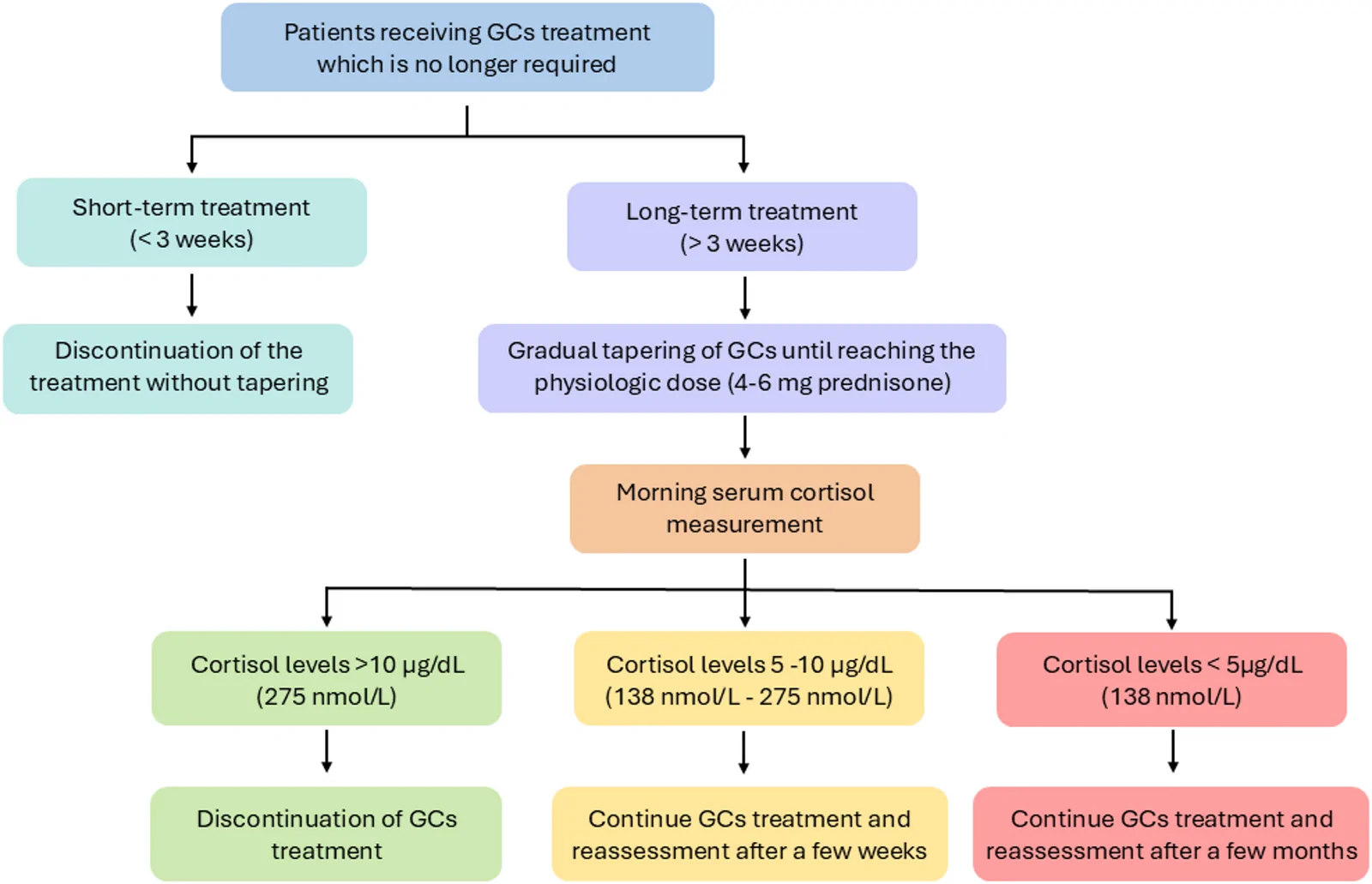
Recommendations for the discontinuation of GCs. The figure presents the strategy for the safe discontinuation of GC therapy once it is no longer indicated. The duration of the treatment determines whether tapering is required. In this case, the dose is gradually tapered until the physiologic dose is reached. Safe discontinuation of GCs can be considered after the assessment of morning serum cortisol levels [8]. (10 µg/dL ≈ 275 nmol/L, 5 µg/dL ≈ 138 nmol/L)

Discontinuation of corticosteroids, especially after long-term use, requires caution. Long-term treatment with GCs (more than 3 weeks) should be gradually tapered until reaching the physiologic dose (4–6 mg prednisone) to prevent worsening of the underlying disease, withdrawal syndrome, or AI. The dose is usually reduced by 10–20% every 1–2 weeks, depending on the duration of prior therapy [8]. According to ESE/ES guidelines [8], when the dose reaches physiologic levels, patients should be evaluated endocrinologically, assessing morning serum cortisol concentrations. The test should be performed by measuring the serum cortisol levels between 8 and 9 a.m., before the morning dose of GCs and at least 24 h after the last dose of treatment. Cortisol levels greater than 10 µg/dL (275 nmol/L) indicate adequate adrenal function, and GCs can be discontinued safely. Cortisol levels lower than 5 µg/dL (138 nmol/L) indicate a high probability of AI, so the patient should continue the treatment and be evaluated after a few months [8]. If cortisol ranges from 5 to 10 µg/dL (138 to 275 nmol/L), there is a high possibility of HPA axis recovery, and for that reason the assessment of morning cortisol should be performed again in a few weeks [8] (Fig. 1). In case of persistent equivocal cortisol concentrations, a dynamic test can be performed [8, 54]. Additionally, it is important to keep in mind that many factors can affect the cortisol levels. Pregnancy and increased estrogen levels can present with elevated cortisol levels, while low albumin with lower serum concentrations [8].

The most common and accessible dynamic test is the ACTH stimulation test. The patient should have stopped GC treatment 24 h before the administration of 250 µg of synthetic ACTH for better accuracy of the test. Stimulated cortisol concentrations depend on the assays used for the test. Indicatively, older assays used a cut-off concentration of 18 µg/dL (500 nmol/L), while newer monoclonal antibody assays and LC-MS/MS have set the cut-off points around 14.5 µg/dL (400 nmol/L) [8, 42, 56]. Concentrations greater than the cut-off indicate normal adrenal function and allow for the discontinuation of the treatment [8, 42].

In case of severe symptoms of withdrawal syndrome occurring after the discontinuation of GCs, a low dose should be restarted, or the dose should be increased to tolerable levels. In short-term treatments, which last less than 3 weeks, GCs can be discontinued without tapering [8].

## Conclusion

The use of GCs in dermatology is extensive. Nevertheless, there are limited practical points addressing the specific challenges of daily dermatological practice. In our review we aimed to provide dermatology-focused practical aspects that were only briefly addressed in the original guidelines about the proper use of GCs. The appropriate use of GCs in dermatology requires a careful balance between therapeutic efficacy and potential adverse effects. Although topical administration is generally considered safe, prolonged application may result in significant systemic absorption and even severe side effects. In addition, the inappropriate or abrupt discontinuation of systemic corticosteroids may lead to AI or adrenal crisis.

To sum up, GCs are valuable therapeutic options, especially for dermatologists, provided that the appropriate drug is selected and administered in individualized doses for the shortest duration possible. Regular clinical monitoring and gradual tapering under endocrinological evaluation, when indicated, should be ensured. Interdisciplinary collaboration between dermatologists and endocrinologists, but also comprehensive patient education, is essential to ensure therapeutic effectiveness while reducing the risk of serious adverse effects.
